# The relevance of including *delirium* in the assessment of sepsis-associated neurological disorders that cause changes in consciousness or confusion

**DOI:** 10.62675/2965-2774.20250211

**Published:** 2025-06-16

**Authors:** Roberta Esteves Vieira de Castro, Yu Kawai, Daniela Nasu Monteiro Medeiros, Arnaldo Prata-Barbosa, Neelima Marupudi

**Affiliations:** 1 Universidade do Estado do Rio de Janeiro Hospital Universitário Pedro Ernesto Department of Pediatrics Rio de Janeiro RJ Brazil Department of Pediatrics, Hospital Universitário Pedro Ernesto, Universidade do Estado do Rio de Janeiro - Rio de Janeiro (RJ), Brazil.; 2 Instituto D’Or de Pesquisa e Ensino Department of Pediatrics Rio de Janeiro RJ Brazil Department of Pediatrics, Instituto D’Or de Pesquisa e Ensino - Rio de Janeiro (RJ), Brazil.; 3 Department of Pediatrics, Division of Pediatric Critical Care Medicine Mayo Clinic Children's Rochester Minnesota United States Department of Pediatrics, Division of Pediatric Critical Care Medicine, Mayo Clinic Children's - Rochester, Minnesota, United States.; 4 Hospital Israelita Albert Einstein Pediatric Intensive Care Unit São Paulo SP Brazil Pediatric Intensive Care Unit, Hospital Israelita Albert Einstein - São Paulo (SP), Brazil.; 5 University of Arizona Banner Children's Hospital Department of Child Health, Division of Pediatric Intensive Care Mesa Arizona United States Department of Child Health, Division of Pediatric Intensive Care, Banner Children's Hospital, University of Arizona - Mesa, Arizona, United States.

## INTRODUCTION

Recently, the Society of Critical Care Medicine (SCCM) Pediatric Sepsis Definition Task Force developed a new, international pediatric sepsis consensus definition, the Phoenix Sepsis Score (PSS). Sepsis and septic shock in children are now diagnosed using objective clinical and laboratory variables across four major organ systems, including the central nervous system (CNS).^(1,2)^ This marks the first time that CNS organ dysfunction has been incorporated into the core definition of pediatric sepsis. The evaluation of neurologic dysfunction in the PSS involves the Glasgow Coma Scale (GCS) and the pupillary reflex test. To develop the PSS, investigators drew on a broad knowledge base, including an international survey, a systematic review, and the analysis of more than 3 million pediatric health consultations, followed by a rigorous consensus process.^(1)^ However, pediatric *delirium* (PD), which is a direct manifestation of CNS organ dysfunction, is not mentioned in the PSS. Numerous terms are used in the literature to describe brain dysfunction during acute disease. Therefore, we aim to show the differences in terminologies and their meanings to standardize language in different clinical practices and research contexts. Furthermore, we want to highlight the importance of evaluating PD in sepsis.

## PEDIATRIC *DELIRIUM*

*Delirium* is a frequent but often underrecognized clinical syndrome of brain dysfunction in septic patients.^(3,4)^ Pediatric *delirium* is independently associated with adverse short- and long-term outcomes, including longer stays in pediatric intensive care units (ICUs) and hospitals, prolonged durations of mechanical ventilation (MV), higher mortality rates, greater direct hospital costs, and long-term cognitive impairment after hospital discharge.^(5,6)^ *Delirium* is widely observed (reported rates of up to 80%) among pediatric patients in critical condition across various disease states.^(4)^ Moreover, the literature shows that PD is present in 63% of septic patients, according to a validated bedside screening tool, the Cornell Assessment of Pediatric *Delirium* (CAP-D).^(6)^

Health care professionals often fail to recognize *delirium* unless screening tools are utilized. The recent PANDEM guidelines from the SCCM recommend the Preschool and Pediatric Confusion Assessment Methods for the ICU (ps/pCAM-ICU) or the CAP-D as the most valid and reliable *delirium* monitoring tools for critically ill pediatric patients.^(4)^ In addition, the European Society of Pediatric and Neonatal Intensive Care (ESPNIC) also recommends the CAP-D as an instrument to assess PD (grade of recommendation = A) and acknowledges the use of the pCAM-ICU and Sophia Observation withdrawal Symptoms-Pediatric *Delirium* scale (SOS-PD).^(7)^ Lastly, the PODIUM Consensus Conference supports a CAP-D score ≥ 9 as an indication of CNS organ dysfunction^(8)^ and the PALICC-2 guideline recommends the use of ps/pCAM-ICU, CAP-D or SOS-PS at least twice daily.^(9)^

The ps/pCAM-ICU is a "point-in-time" assessment tool derived from the highly reliable Confusion Assessment Method for the ICU (CAM-ICU) and adapted for pediatric patients. The pCAM-ICU, validated in patients over 5 years old, has high sensitivity (83%) and specificity (99%), with excellent interrater reliability (kappa [κ] = 0.96). The psCAM-ICU, adapted for children under 5 years of age, has good sensitivity (75%) and high specificity (91%), with reliability (κ = 0.79). It has also been validated in infants younger than 6 months, showing a sensitivity of 95% and specificity of 81%. The CAP-D demonstrated high sensitivity (94%) and specificity (79%) in a mixed medical-surgical pediatric ICU population, with strong interrater reliability among nursing staff (κ = 0.94), although reliability is lower in children under 2 years old (κ = 0.6).^(4,10,11)^ Finally, the SOS-PD scale has an overall sensitivity of 92.3% and a specificity of 96.5%.^(12)^

**Figure 1:**
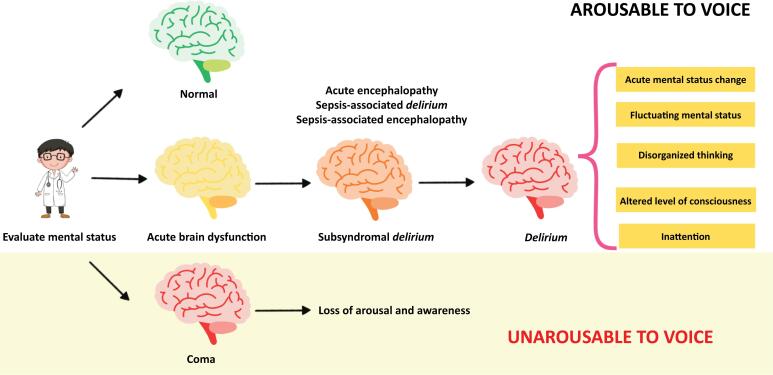
Recommended nomenclature for describing acute cognitive disturbances in clinical practice.

The use of the term "acute encephalopathy" is discouraged when describing clinical features observed at the bedside.^(5)^ Instead, Slooter et al. suggest using "subsyndromal *delirium*" to describe acute cognitive changes that resemble *delirium* but do not meet all the gold standard criteria outlined in the Diagnostic and Statistical Manual of Mental Disorders, fifth edition text revision (DSM-5-TR).^(5,13)^ The term "*delirium*" should be reserved for cases that meet DSM-5-TR criteria, whereas "coma" should describe a state of severely reduced responsiveness, as defined by diagnostic tools such as the GCS or the Full Outline of UnResponsiveness (FOUR) score^(5)^ (Figure 1). Importantly, prior coma is a nonmodifiable risk factor that has been strongly linked to *delirium*.^(14)^

– **Acute encephalopathy:** A rapidly developing (less than four weeks but usually within hours to a few days) pathobiological process in the brain that may present as subsyndromal *delirium*, *delirium*, or, in the case of a severely decreased level of consciousness, coma, all of which represent a change from baseline cognitive status.
– **Coma:** A clinical state of severely depressed responsiveness, recognized by diagnostic instruments such as the Glasgow Coma Scale or the Full Outline of Unresponsiveness score.
– ***Delirium***: According to the DSM-5-TR, *delirium* is described if criteria A to E are met:
  - Disturbance in attention and awareness.
  - The disturbance develops over a short period of time (usually hours to a few days), represents a change from baseline attention and awareness, and tends to fluctuate in severity throughout the day.
  - Presence of additional cognitive disturbance.
  - The disturbances in criteria A and C are not explained by another preexisting, established, or evolving neurocognitive disorder and do not occur in the context of a severely reduced level of arousal (e.g., coma).
  - Evidence from history, physical examination, or laboratory findings indicates that the disturbance is a direct physiologic consequence of another medical condition, substance intoxication or withdrawal, toxin exposure, or multiple etiologies.
– **Subsyndromal *delirium*:** Clinical conditions resembling delirium are marked by disruptions in attention, executive functioning, and circadian rhythm, and the degree of cognitive impairment does not meet the full diagnostic threshold for *delirium*.
– **Sepsis-associated *delirium*:** Brain manifestations that commonly occur in septic patients and are believed to develop due to a combination of neuroinflammation and disturbances in cerebral perfusion, the blood–brain barrier, and neurotransmission.
– **Sepsis-associated encephalopathy:** Diffuse brain dysfunction not caused by a specific central nervous system infection and clinically manifested as a disturbance of consciousness, with changes in perception similar to *delirium*. Additional symptoms may include irritability, varying degrees of coma, and, more rarely, convulsions, tremors, asterixis, or myoclonus.

Sources: Adapted from the American Psychiatry Association, 2022^(13)^; Atterton et al., 2020^(3)^; Slooter et al. 2020^(5)^; De Araújo et al., 2022^(6)^.

## WHY THE GLASGOW COMA SCALE FALLS SHORT IN ASSESSING ACUTE BRAIN DYSFUNCTION

The GCS is typically represented as a single score combining three assessments: eye opening, verbal response, and motor response. Although extensively researched and integrated into various scoring systems, the GCS has shown variable interrater reliability. Studies have reported a broad range of κ scores, with one study noting values ranging from 0.39 - 0.79. Discrepancies in scoring are more common between different professional groups (e.g., nurses *versus* medical doctors), particularly regarding the motor score, with higher disagreement rates observed among less experienced staff and patients with intermediate scores. Conversely, the lowest levels of disagreement are found within specialized professional groups (e.g., neurocritical care nurses), particularly when evaluating the verbal component or assessing patients who are alert or slightly drowsy.^(15)^

Recently, Sanchez-Pinto et al. evaluated sepsis-associated encephalopathy in children using the qSOFA score, which uses the GCS to determine mental state.^(2)^ Cheung et al. also used the GCS to diagnose disorders of consciousness in pediatric patients with severe sepsis (GCS score < 12 with no sedatives).^(16)^ However, the GCS lacks key elements for assessing crucial components of *delirium*, such as inattention, purposefulness, restlessness, and consolability, making it unsuitable for evaluating this condition.^(5,6)^ For example, a child with a GCS score ≥ 12, who may not have been classified as having encephalopathy in previous studies, can still experience *delirium*.^(17)^ Additionally, the GCS is difficult to interpret in patients receiving invasive MV, which is a common clinical scenario in septic shock. Additionally, a low GCS score may indicate the effects of interventions related to critical illness, such as sedation or neuromuscular blockade, rather than directly reflecting primary organ dysfunction.^(2)^

## CONCLUSIONS

Consistently, bedside screening is essential to prevent the underestimation of *delirium* incidence, as its fluctuating nature must be considered. We strongly believe that diagnosing *delirium* using bedside tools as a marker of central nervous system dysfunction is superior to diagnosing "encephalopathy" via the Glasgow Coma Scale. Prompt recognition of pediatric *delirium* enables timely *delirium* management. Additionally, it helps identify vulnerable patients with a sepsis phenotype who require additional care to reduce the risk of developing pediatric *delirium* and supports the identification of an important cohort for future research on adjuvant therapies and prognosis.

Finally, we emphasize that *delirium* assessment should be a standard part of daily pediatric intensive care unit care. Including this recommendation in pediatric sepsis management will improve quality of care, facilitate timely management, and reduce the risk of negative short- and long-term outcomes. *Delirium* evaluation is part of a new era in pediatric intensive care medicine.

## References

[1] Schlapbach LJ, Watson RS, Sorce LR, Argent AC, Menon K, Hall MW (2024). Society of Critical Care Medicine Pediatric Sepsis Definition Task Force. International Consensus Criteria for Pediatric Sepsis and Septic Shock. JAMA.

[2] Sanchez-Pinto LN, Bennett TD, Stroup EK, Luo Y, Atreya M, Bubeck Wardenburg J (2023). Derivation, validation, and clinical relevance of a pediatric sepsis phenotype with persistent hypoxemia, encephalopathy, and shock. Pediatr Crit Care Med.

[3] Atterton B, Paulino MC, Povoa P, Martin-Loeches I (2020). Sepsis associated delirium. Medicina (Kaunas).

[4] Smith HA, Besunder JB, Betters KA, Johnson PN, Srinivasan V, Stormorken A (2022). 2022 Society of Critical Care Medicine Clinical Practice Guidelines on Prevention and Management of Pain, Agitation, Neuromuscular Blockade, and Delirium in Critically Ill Pediatric Patients with Consideration of the ICU Environment and Early Mobility. Pediatr Crit Care Med.

[5] Slooter AJ, Otte WM, Devlin JW, Arora RC, Bleck TP, Claassen J (2020). Updated nomenclature of delirium and acute encephalopathy: statement of ten Societies. Intensive Care Med.

[6] de Araújo BE, da Silva Fontana R, de Magalhães-Barbosa MC, Lima-Setta F, Paravidino VB, Riveiro PM (2022). Clinical features, electroencephalogram, and biomarkers in pediatric sepsis-associated encephalopathy. Sci Rep.

[7] Harris J, Ramelet AS, van Dijk M, Pokorna P, Wielenga J, Tume L (2016). Clinical recommendations for pain, sedation, withdrawal and delirium assessment in critically ill infants and children: an ESPNIC position statement for healthcare professionals. Intensive Care Med.

[8] Wainwright MS, Guilliams K, Kannan S, Simon DW, Tasker RC, Traube C (2022). Pediatric Organ Dysfunction Information Update Mandate (PODIUM) Collaborative. Acute neurologic dysfunction in critically ill children: The PODIUM Consensus Conference. Pediatrics.

[9] Valentine SL, Kudchadkar SR, Ward S, Morrow BM, Nadkarni VM, Curley MAQ, Second Pediatric Acute Lung Injury Consensus Conference (PALICC-2) of the Pediatric Acute Lung Injury and Sepsis Investigators (PALISI) Network (2023). Nonpulmonary Treatments for Pediatric Acute Respiratory Distress Syndrome: From the Second Pediatric Acute Lung Injury Consensus Conference. Pediatr Crit Care Med.

[10] Canter MO, Tanguturi YC, Ellen Wilson J, Williams SR, Exum SA, Umrania HM (2021). Prospective validation of the preschool confusion assessment Method for the ICU to screen for delirium in infants less than 6 months old. Crit Care Med.

[11] Barbosa MD, Andrade LB, Duarte MD, Castro RE (2023). Translation and cross-cultural adaptation of the anchor points of the Cornell Assessment of Pediatric Delirium scale into Portuguese. Crit Care Sci.

[12] Ista E, van Beusekom B, van Rosmalen J, Kneyber MC, Lemson J, Brouwers A (2018). Validation of the SOS-PD scale for assessment of pediatric delirium: a multicenter study. Crit Care.

[13] American Psychiatric Association (2022). Diagnostic and Statistical Manual of Mental Disorders.

[14] Devlin JW, Skrobik Y, Gélinas C, Needham DM, Slooter AJ, Pandharipande PP (2018). Clinical Practice Guidelines for the Prevention and Management of Pain, Agitation/Sedation, Delirium, Immobility, and Sleep Disruption in Adult Patients in the ICU. Crit Care Med.

[15] Riker RR, Fugate JE (2014). Participants in the International Multi-disciplinary Consensus Conference on Multimodality Monitoring. Clinical monitoring scales in acute brain injury: assessment of coma, pain, agitation, and delirium. Neurocrit Care.

[16] Cheung C, Kernan KF, Berg RA, Zuppa AF, Notterman DA, Pollack MM (2023). on behalf of the Eunice Kennedy Shriver National Institute of Child Health and Human Development Collaborative Pediatric Critical Care Research Network. Acute disorders of consciousness in pediatric severe sepsis and organ failure: secondary analysis of the multicenter phenotyping sepsis-induced multiple organ failure study. Pediatr Crit Care Med.

[17] Hamed SA, Hamed EA, Abdella MM (2009). Septic encephalopathy: relationship to serum and cerebrospinal fluid levels of adhesion molecules, lipid peroxides and S-100B protein. Neuropediatrics.

